# Two year persistent treatment effect in reducing nasal symptoms of cat allergy after 4 doses of Cat-PAD, the first in a new class of synthetic peptide immuno-regulatory epitopes

**DOI:** 10.1186/2045-7022-3-S2-O7

**Published:** 2013-07-16

**Authors:** Roderick Hafner, Peter Couroux, Kristen Armstrong, Deepen Patel, Mark Larche, Brett Haumann

**Affiliations:** 1 Circassia Ltd, R&D, Oxford, UK; 2 Cetero Research, R&D, Missisauga, Canada; 3 Adiga, Life Sciences, Hamilton, Canada; 4 Cetero Research, R&D, Toronto, Canada; 5 McMaster University, Department of Medicine, Hamilton, Canada

## Background

Treatment with Cat-PAD (also known as ToleroMune® Cat, the first in a new class of synthetic peptide immuno-regulatory epitopes), in an Environmental Exposure Chamber (EEC) model of cat allergy showed a persistent treatment effect one year [1] and two years [2] after administration of only 4 injections over 12 weeks. Here we report the differences in Total Nasal Symptom Scores (TNSS) between Cat-PAD treatment arms and placebo two years after treatment started.

## Method

Originally 202 subjects were randomised to 4x6nmol Cat-PAD 4 weeks (wk) apart, 8x3nmol Cat-PAD 2wk apart, or placebo. EEC challenges were performed at baseline and 18-22wk. 89 subjects were recruited into a follow-on study one year after the start of treatment. Of the subjects who completed the one year EEC challenge, 50 subjects were recruited for a further EEC challenge 100-104wk after the start of treatment. All EEC challenges consisted of 4 consecutive days of 3 hours (h) of allergen exposure with Fel d1 levels of circa 50 ng/m3. The 4 day challenge was designed to ensure late phase responses were present in the nasal airway. Subjects scored each of 4 symptoms (Running nose; Sneezing; Blocked nose; Itchy nose) on a scale of 0-3 every 30 minutes during the EEC challenge. These scores were summed to give a TNSS on a scale of 0-12.

## Results

The least squares (LS) mean TNSS was significantly lower (p <0.05) for 4x6nmol Cat-PAD vs placebo at the following EEC challenge: Day 1 at 2h and 2.5h; Day 2 at 2h, 2.5h and 3h; Day 3 at 1h, 1.5h, 2h, 2.5h and 3h. On Day 4 of EEC Challenge, when the cumulative allergen challenge is greatest and late phase responses in the nose are likely to be maximal, LS mean TNSS was significantly lower for 4x6nmol Cat-PAD vs placebo at 2h (4.818 vs 7.762, p=0.0090), 2.5h (5.091 vs 7.667, p=0.0290) and 3h (4.818 vs 7.952, p=0.0080) time points. No significant reductions in LS mean TNSS were observed for 8x3nmol Cat-PAD vs placebo.

## Conclusion

Treatment with 4 injections of 6nmol Cat-PAD over 12wk showed a substantial reduction in patients' TNSS that persisted two years after starting treatment. The treatment effect is substantial under conditions where late phase responses are expected to be present in the nasal airway. Cat-PAD is the first in a new class of synthetic peptide immuno-regulatory epitopes and may confer long-term disease-modification in chronic nasal airway disease due to cat allergy.
